# The Furnishing and Appliances of a Cottage Hospital

**Published:** 1897-04-24

**Authors:** 


### The Institutional Workshop.

### THE FURNISHING AND APPLIANCES OF A COTTAGE HOSPITAL.

We have frequently been asked for full details and cost of furnishing a cottage hospital, and have therefore pleasure in publishing the following list of the contents of the Tilbury Cottage Hospital, as supplied to us by the Hon. Sydney Holland. In Mr. Burdett's book on "Cottage Hospitals" a large amount of particulars are given, but nowhere else that we are aware of can a list such as we publish below be procured :—

### Contents of Four Wards (Eight Beds).

| Quantity. | | £ | s. | d. |
|---|---|---|---|---|
| 8 | Bedsteads (spring, head-piece made to let down) with chain pulleys, at £2 5s. 6d.... | 18 | 4 | 0 |
| 8 | Hair mattresses, at £1 9s. 6d. ... ... | 11 | 16 | 0 |
| 8 | Feather bolsters, at 5s. 9d. ... ... ... | 2 | 6 | 0 |
| 8 | Feather pillows, at 4s. 6d. ... ... ... | 1 | 16 | 0 |
| 6 | Hair pillows, at 4s. 6d. ... ... ... | 1 | 7 | 0 |
| 8 | Lockers (ashwood, with marble tops) at £1 14s 6d. ... ... ... ... ... | 13 | 16 | 0 |
| 8 | Bentwood chairs (walnut), at 3s. 6d. ... | 1 | 8 | 0 |
| 6 | Windsor chairs, at 4s. 6d. ... ... ... | 1 | 7 | 0 |
| 2 | Windsor armchairs, at 9s. 6d. ... ... | 0 | 19 | 0 |
| 4 | Cane chairs (low, with sloping seats, for nurses), at 9s. 6d. ... ... ... | 1 | 18 | 0 |
| 4 | Screens (4-fold bamboo), at £1 6s. ... ... | 5 | 4 | 0 |
| 4 | Turkey twill curtains for screens, complete with tapes, &c. ... ... ... ... | 2 | 19 | 9 |
| 2 | Ashwood tables, with marble tops and three cupboards fitted into recess, at £9 2s. 6d. | 18 | 5 | 0 |
| 2 | Deal tables, 3 ft. 6 in. by 2 ft. 3 in., at 20s. | 2 | 0 | 0 |
| 2 | Deal tables, 3 ft. by 2 ft., at 17s. ... ... | 1 | 14 | 0 |
| 3 | Waggon tables on casters, with marble tops and shelf underneath, at £2 17s. 6d. ... | 8 | 12 | 6 |
| 1 | Cupboard in recess of one ward ... ... | 4 | 2 | 6 |
| 8 | Tin cases (for temperature charts to hang on head of bedstead), at 8d. ... ... | 0 | 5 | 4 |
| 8 | Larger size tin cases (for admission paper), at 1s. ... ... ... ... ... ... | 0 | 8 | 0 |
| | | £98 | 8 | 1 |

### Operating-room and Dispensary.

| Quantity. | | £ | s. | d. |
|---|---|---|---|---|
| 1 | Operating table (painted deal, movable leg pieces, mattress, macintosh ... ... | 6 | 6 | 0 |
| | Repairing and fitting an old surgical instrument case (given by Poplar Hospital Committee) ... ... ... ... | 2 | 12 | 6 |
| 1 | Painted cupboard for dispensary, fitted with shelves and drawers, inclosed cupboards underneath ... ... ... ... | 10 | 18 | 0 |
| 2 | Marble slabs for windows ... ... ... | 1 | 2 | 0 |
| 31 yds | Linoleum, at 2s. 7d. ... ... ... ... | 4 | 0 | 1 |
| | | £24 | 18 | 7 |

### Kitchen and Scullery.

| Quantity. | | £ | s. | d. |
|---|---|---|---|---|
| 1 | Kitchen table ... ... ... ... | 1 | 11 | 0 |
| 3 | Windsor chairs, at 4s. 6d. ... ... ... | 0 | 13 | 6 |
| 18⅓ yds | Oil cloth, at 3s. 3d. ... ... ... ... | 2 | 19 | 7 |
| | | £5 | 4 | 1 |

### Sundries.

| Quantity. | | £ | s. | d. |
|---|---|---|---|---|
| 31 yds | Green union blinds (complete) ... ... | 11 | 8 | 6 |
| | Expenses to measure carpets, blinds ... | 0 | 2 | 0 |
| 1 | Umbrella stand ... ... ... ... | 0 | 12 | 6 |
| 2 | Cork mats (for bath-rooms) ... ... ... | 0 | 3 | 6 |
| 1 | Cocoa mat (front entrance) ... ... ... | 0 | 13 | 0 |
| 1 | Cocoa mat (back entrance) ... ... ... | 0 | 7 | 9 |
| 55⅔ yds | Linoleum (corridors), at 2s. 6d. ... ... | 6 | 19 | 2 |
| 17 yds | Linoleum (stairs), at 2s. 0½d. ... ... | 1 | 14 | 8½ |
| | Laying same ... ... ... ... ... | 0 | 16 | 0 |
| | Refrigerator ... ... ... ... ... | 3 | 12 | 0 |
| | Set of account books ... ... ... ... | 4 | 3 | 6 |
| | Portable fire hand-pump ... ... ... | 4 | 16 | 9 |
| 6 | Fire buckets, red japanned, and lettered "FIRE" ... ... ... ... ... ... | 1 | 4 | 0 |

### Sundries (*continued*).

| Quantity. | | £ | s. | d. |
|---|---|---|---|---|
| 12 | Hand grenades and 4 baskets ... ... | 2 | 0 | 6 |
| | Dust bin ... ... ... ... ... | 0 | 6 | 9 |
| | Scraper, with brush ... ... ... ... | 0 | 10 | 6 |
| | | £39 | 11 | 1½ |

### Matron's Sitting-room.

| Quantity. | | £ | s. | d. |
|---|---|---|---|---|
| 1 | Book case for recess ... ... ... ... | 5 | 3 | 6 |
| 1 | Bamboo writing table ... ... ... | 3 | 12 | 6 |
| 2 | Rush seat chairs at 6s. 9d. ... ... ... | 0 | 13 | 6 |
| 1 | Wicker chair (cushioned) ... ... ... | 1 | 3 | 6 |
| 1 | Lounge (tapestry) ... ... ... ... | 6 | 0 | 0 |
| 1 | Mahogany occasional table ... ... ... | 1 | 2 | 0 |
| 1 | White overmantel ... ... ... ... | 2 | 5 | 0 |
| 21 yds | Kalmuck carpet (resida green) at 2s. 8d. ... | 2 | 16 | 0 |
| | Making same ... ... ... ... | 0 | 5 | 3 |
| 48 yds | Lincrusta paper (white panelled) ... ... | 4 | 16 | 0 |
| | Supplying and fixing china shelves, with shaped brackets and mouldings to top of high dado, panelling walls, and painting shelves white ... ... ... | 18 | 17 | 6 |
| 1 | Brass rod (11 ft.) ... ... ... ... | 0 | 8 | 8½ |
| | Rings and hooks, &c.... ... ... ... | 0 | 7 | 6 |
| 1 pr. | White curtains, with loops and rings ... | 0 | 17 | 6 |
| 3 prs. | Serge curtains, with hooks, &c. ... ... | 2 | 17 | 9 |
| | | £51 | 6 | 2½ |

### Nurses' Sitting-room.

| Quantity. | | £ | s. | d. |
|---|---|---|---|---|
| 1 | Dining table (4 by 3) ... ... ... ... | 1 | 3 | 6 |
| 1 | Folding chair ... ... ... ... ... | 0 | 6 | 6 |
| 1 | Wicker chair (cushioned) ... ... ... | 1 | 3 | 6 |
| 3 | Rush seat chairs at 6s. 9d, ... ... ... | 1 | 0 | 3 |
| 1 | Painted hanging bookshelf ... ... ... | 1 | 9 | 6 |
| 1 | Corner cupboard ... ... ... ... | 2 | 19 | 0 |
| 12 yds | Cork carpet (resida green) at 3s. ... ... | 1 | 16 | 0 |
| 1 | Brass rod (ends, brackets, hooks) ... ... | 0 | 11 | 3 |
| 1 | White overmantel ... ... ... ... | 2 | 5 | 0 |
| | | £12 | 14 | 6 |

### Matron's Bed-room.

| Quantity. | | £ | s. | d. |
|---|---|---|---|---|
| 1 | Bedstead and spring ... ... ... ... | 1 | 1 | 0 |
| 1 | Hair mattress ... ... ... ... ... | 1 | 9 | 6 |
| 1 | Feather bolster ... ... ... ... | 0 | 5 | 9 |
| 1 | Feather pillow... ... ... ... ... | 0 | 4 | 6 |
| 1 | Painted wardrobe to fit recess ... ... | 5 | 0 | 0 |
| 1 | Satin walnut dressing table, 1 washstand, 2 chairs, linen basket ... ... ... | 5 | 5 | 0 |
| | Brass rods, ends, loophooks, rings ... ... | 0 | 10 | 6 |
| 1 | Toilet pail ... ... ... ... ... | 0 | 3 | 0 |
| | | £13 | 19 | 3 |

### Three Nurses' Bed-rooms.

| Quantity. | | £ | s. | d. |
|---|---|---|---|---|
| 3 | Bedsteads and springs at 21s. ... ... ... | 3 | 3 | 0 |
| 3 | Hair mattresses at 29s. 6d. ... ... ... | 4 | 8 | 6 |
| 3 | Feather bolsters at 5s. 9d. ... ... ... | 0 | 17 | 3 |
| 3 | Feather pillows at 4s. 6d. ... ... ... | 0 | 13 | 6 |
| 3 | Satin walnut bed-room suites (including wardrobe, washstand, dressing table with three drawers large and small, two chairs, linen basket, at £7 10s. ... ... | 22 | 10 | 0 |
| 16 yds | Kalmuck carpet (strips) at 2s. 8d. ... ... | 2 | 2 | 8 |
| 6 | Brass dress hooks ... ... ... ... | 0 | 5 | 3 |
| 3 | Toilet pails ... ... ... ... ... | 0 | 9 | 0 |
| | | £34 | 9 | 2 |

### Servants' Bed-room (for Two).

| Quantity. | | £ | s. | d. |
|---|---|---|---|---|
| 2 | Bedsteads (spring) ... ... ... ... | 2 | 2 | 0 |
| 2 | Mattresses, bolsters, and pillows ... ... | 3 | 19 | 6 |
| 2 | Japanned chests of drawers at £1 4s. ... | 2 | 8 | 0 |
| 2 | Japanned washstands at 7s. 6d. ... ... | 0 | 15 | 0 |
| 2 | Japanned towel rails at 2s. 3d. ... ... | 0 | 4 | 6 |
| 2 | Chairs at 3s. ... ... ... ... ... | 0 | 6 | 0 |
| 2 | Japanned dressing glasses at 5s. ... ... | 0 | 10 | 0 |
| | | £10 | 5 | 0 |

### Linen.

#### Wards (8 beds).

| Quantity. | | £ | s. | d. |
|---|---|---|---|---|
| 16 | Counterpanes at 6s. 6d. | 5 | 4 | 0 |
| 8 prs. | Blankets (white), at 8s. | 3 | 4 | 0 |
| 4 ,, | Blankets (brown), at 6s. 9d. | 1 | 7 | 0 |
| 24 ,, | Sheets (twill cotton), at 2s. 9d. | 8 | 2 | 0 |
| 36 | Draw-sheets (twill cotton), at 2s. 9d. | 4 | 19 | 0 |
| 24 | Pillow slips, at 8½d. | 0 | 17 | 0 |
| 12 | Towels (for patients) | 0 | 7 | 0 |
| 12 | Towels (for doctors' use—huckaback) | 0 | 9 | 0 |
| 12 | Tea cloths | 0 | 6 | 3 |
| 6 | Table cloths, at 2s. 9d. | 0 | 16 | 6 |
| 12 | Lavatory cloths | 0 | 4 | 9 |
| | | £25 | 16 | 6 |

#### Staff (4 beds).

| Quantity. | | £ | s. | d. |
|---|---|---|---|---|
| 6 | Quilts (white), at 7s. | 2 | 2 | 0 |
| 8 prs. | Sheets, at 6s. 9d. | 2 | 14 | 0 |
| 4 ,, | Blankets (white), at 8s. | 1 | 12 | 0 |
| 2 ,, | Blankets (brown under), at 6s. 9d. | 0 | 13 | 6 |
| 12 | Pillow slips, at 8½d. | 0 | 8 | 6 |
| 6 | Toilets (white), at 10½d. | 0 | 5 | 3 |
| 12 | Towels (huckaback) | 0 | 9 | 6 |
| 12 | Towels (bath) | 0 | 17 | 6 |
| 4 | Table cloths, at 8s. 3d. | 1 | 13 | 0 |
| 12 | Serviettes | 0 | 8 | 3 |
| 12 | Dusters | 0 | 4 | 0 |
| 18 | Roller towels, at 20s. doz. | 1 | 10 | 0 |
| | | £12 | 17 | 6 |

#### Servants (two beds).

| Quantity. | | £ | s. | d. |
|---|---|---|---|---|
| 3 | Quilts, at 3s. 3d. | 0 | 9 | 9 |
| 2 prs. | Blankets (brown), at 6s. 9d. | 0 | 13 | 6 |
| 1 pr. | Blankets (under) | 0 | 5 | 6 |
| 4 prs. | Sheets, at 6s. 9d. | 1 | 7 | 0 |
| 6 | Pillow-slips, at 8½d. | 0 | 4 | 3 |
| 6 | Towels (honeycomb), at 6s. 3d. doz. | 0 | 3 | 1½ |
| 4 | Toilets, at 1s. 0½d. | 0 | 4 | 2 |
| 3 | Tablecloths (kitchen), at 2s. 9d. | 0 | 8 | 3 |
| 12 | Teacloths (kitchen) | 0 | 6 | 3 |
| | | £4 | 1 | 9½ |

### China and Glass.

| Quantity. | | £ | s. | d. |
|---|---|---|---|---|
| 4 | Toilet sets (nurses), at 5s. 11d. | 1 | 3 | 8 |
| 2 | Toilet sets (servants), at 5s. | 0 | 10 | 0 |
| 6 | Bottles and tumblers, at 7½d. | 0 | 3 | 9 |
| 1 | Dinner set | 1 | 1 | 0 |
| 1 doz. | Soup plates | 0 | 3 | 6 |
| 1 | Soup tureen | 0 | 8 | 0 |
| 1 | Tea set | 0 | 16 | 9 |
| 6 | Breakfast cups and saucers | 0 | 5 | 6 |
| 1 | Tea-pot | 0 | 2 | 3 |
| 1 doz. | Cups and saucers (for patients) | 0 | 6 | 6 |
| 1 ,, | Tumblers (for patients) | 0 | 2 | 6 |
| 1 ,, | Tumblers (for patients) | 0 | 4 | 3 |
| 1 | Glass jug | 0 | 1 | 10 |
| 1 set | Jugs | 0 | 1 | 11 |
| 1 | Cruet | 0 | 2 | 6 |
| 1 set | Pie-dishes | 0 | 1 | 5½ |
| 6 | Bowls | 0 | 2 | 9 |
| 1 set | Basins | 0 | 0 | 8 |
| 1 set | Pans (brown) | 0 | 3 | 1 |
| 1 doz. | Plates (large, for patients) | 0 | 3 | 6 |
| 1 ,, | Plates (small, ,, ) | 0 | 2 | 9 |
| 3 | Dishes (white, ,, ) | 0 | 5 | 7 |
| 2 | White ewers and basins (for doctors' use in wards) | 0 | 7 | 0 |
| | | £7 | 0 | 8½ |

### Ironmongery, &c.

| Quantity. | | £ | s. | d. |
|---|---|---|---|---|
| 1 | Tea tray | 0 | 3 | 3 |
| 1 | Spice box | 0 | 2 | 10 |
| 1 | Paste board and pin | 0 | 2 | 7 |
| 1 | Colander | 0 | 2 | 1 |
| 1 | Egg whisk | 0 | 0 | 5 |
| 1 | Corkscrew | 0 | 0 | 10 |
| 1 | Teapot (metal, for wards) | 0 | 5 | 0 |
| 1 | Slop pail | 0 | 4 | 9 |
| 4 | Coal scuttles (2 at 4s. 6d., 2 at 3s. 8d.) | 0 | 16 | 4 |
| 3 | Hair brooms at 3s. | 0 | 9 | 0 |
| 3 | Dust pan brushes at 2s. | 0 | 6 | 0 |
| 1 | Whisk brush | 0 | 2 | 0 |

### Ironmongery, &c. (*continued*).

| Quantity. | | £ | s. | d. |
|---|---|---|---|---|
| 6 | Brass scrubs at 9d. | 0 | 4 | 6 |
| 6 | Stove brushes at 1s. 10d. | 0 | 11 | 0 |
| 6 | ,, round | 0 | 2 | 0 |
| 1 set | Shoe brushes | 0 | 5 | 6 |
| 6 | Saucepan brushes | 0 | 2 | 9 |
| 6 | Mops | 0 | 2 | 0 |
| 2 | Pails (galvanized) | 0 | 2 | 4 |
| 3 | Pie dishes (enamelled) | 0 | 2 | 3 |
| 1 | Bread board | 0 | 1 | 4 |
| 1 | Knife (bread) | 0 | 1 | 6 |
| 1 doz. | Table knives and forks (patients) | 0 | 14 | 0 |
| 1 ,, | Small knives and forks (patients) | 0 | 12 | 0 |
| 1 pr. | Carvers | 0 | 2 | 9 |
| 1 | Steel | 0 | 1 | 1 |
| ½ doz. | Table knives (staff) | 0 | 12 | 0 |
| ½ ,, | Small knives (staff) | 0 | 10 | 0 |
| 1 pr. | Carvers (staff) | 0 | 9 | 6 |
| 2 | Tin kettles, at 1s. 6d. | 0 | 3 | 0 |
| 1 | Iron kettle | 0 | 6 | 0 |
| 1 | Boiling pot | 0 | 15 | 0 |
| 6 | Iron saucepans | 1 | 3 | 5 |
| 1 | Milk saucepan | 0 | 3 | 8 |
| 2 | Frying pans, at 3s. 6d. and 1s. | 0 | 4 | 6 |
| 1 | Fish kettle | 0 | 4 | 8 |
| 1 | Fish slice | 0 | 0 | 10 |
| 1 | Half-pint soup ladle | 0 | 1 | 3 |
| 1 set | Weights and scales | 0 | 12 | 9 |
| 4 | Tin-plated covers, at 1s. | 0 | 4 | 0 |
| 1 | Mincing machine | 0 | 6 | 0 |
| 1 | Meat chopper | 0 | 1 | 4 |
| 1 | Meat saw | 0 | 1 | 6 |
| 1 | Flour dredger | 0 | 0 | 8 |
| 2 | Hot-water bottles (tin, for wards), at 3s. 9d. | 0 | 7 | 6 |
| 1 | Sugar dredger | 0 | 1 | 0 |
| 1 | Salt box | 0 | 1 | 4 |
| 2 | Tin funnels | 0 | 0 | 4 |
| 1 | Gravy strainer | 0 | 0 | 9½ |
| 1 | Bread grater | 0 | 0 | 6½ |
| 1 | Knife tray | 0 | 2 | 2 |
| 1 | Set skewers | 0 | 0 | 7 |
| 1 | Toast fork | 0 | 0 | 4 |
| 1 | Tea tray | 0 | 2 | 9 |
| ½ doz. | Plated table forks (staff) at 26s. | 0 | 13 | 0 |
| ½ ,, | Plated small forks (staff) at 19s. | 0 | 9 | 6 |
| 4 ,, | Plated table spoons (staff) at 2s. 2d. | 0 | 8 | 8 |
| ½ ,, | Plated dessert spoons (staff) at 19s. | 0 | 9 | 6 |
| ½ ,, | Plated tea spoons (staff) at 11s. 6d. | 0 | 5 | 9 |
| 1 ,, | Nickel-plated dessert spoons (patients) | 0 | 4 | 6 |
| 1 ,, | Nickel-plated tea spoons (patients) | 0 | 2 | 2 |
| 4 | Iron spoons | 0 | 1 | 0 |
| 1 | Mahogany coal-box (matron's room) | 0 | 18 | 0 |
| 1 | Japanned coal-box (nurses' room) | 0 | 8 | 0 |
| 2 | Tin dish covers (large and small) at 8s. 9d. and 4s. | 0 | 12 | 9 |
| 12 | Door stops | 0 | 5 | 6 |
| 11 | Black kerb fenders (wards and all rooms), at 9s. | 4 | 19 | 0 |
| 1 | Kitchen fender | 0 | 10 | 0 |
| 7 | Pokers | 0 | 14 | 0 |
| 6 sets | Fireirons, at 4s. 6d. | 1 | 7 | 0 |
| 1 | Kitchen set | 0 | 4 | 6 |
| 2 | Hot-water cans, at 2s. | 0 | 4 | 0 |
| 1 | Flue brush | 0 | 2 | 0 |
| 2 | Dustpans | 0 | 2 | 2 |
| | | £25 | 18 | 6 |

### China and Enamelled Goods for Wards.

| Quantity. | | £ | s. | d. |
|---|---|---|---|---|
| ½ doz. | Enamelled basins (for carbolic lotion) | 0 | 4 | 3 |
| 2 ,, | Enamelled jugs, at 4s. 6d. and 1s. 8d. | 0 | 6 | 2 |
| 4 ,, | Enamelled bowls and saucers (small) | 0 | 2 | 6 |
| 2 | Enamelled "kidney" receivers | 0 | 12 | 8 |
| 1 | Enamelled irrigator | 0 | 7 | 0 |
| 6 | Enamelled spittoons, at 18s. | 0 | 9 | 0 |
| 1 | Enamelled pail (for soiled dressings) | 0 | 7 | 0 |
| 6 | Porcelain feeders | 0 | 4 | 0 |
| 4 | Porcelain dressing bowls, at 7½d. | 0 | 2 | 6 |
| 2 | Porcelain graduated porringers, at 2s. 6d. | 0 | 5 | 0 |
| 3 | Porcelain dressing trays, at 1s. 4d., 2s., 7s. | 0 | 10 | 4 |
| 3 | Porcelain triangular receivers, at 2s. | 0 | 6 | 0 |
| 2 | Glass graduated measures, 10 oz. 10d., 20 oz. 1s. 3d. | 0 | 2 | 1 |

China and Enamelled Goods for Wards (*continued*).

| Quantity. | | £ | s. | d. |
|---|---|---|---|---|
| 6 | Glass tubes (for specimens) at 5½d.... ... | 0 | 2 | 9 |
| 2 | Glass syringes ... ... ... ... ... | 0 | 2 | 6 |
| 1 | Glass covered jar (for drainage tubes) ... | 0 | 4 | 0 |
| 2 | White jars and covers (for sponges) ... | 0 | 4 | 0 |
| 3 | Stone bottles, 1 gall. (for antiseptic lotions), at 10½d. ... ... ... ... ... ... | 0 | 2 | 7½ |
| 6 | Medicine glasses (2 oz.), at 7d. ... ... | 0 | 3 | 6 |
| 3 | Plain stoppered bottles (for sutures, &c.), at 8d.... ... ... ... ... ... | 0 | 2 | 0 |
| 6 | Glass urinals, at 1s. 0½d. ... ... ... | 0 | 6 | 3 |
| 6 | Ointment jars (1 lb.), at 10d. ... ... | 0 | 5 | 0 |
| 3 | Ointment jars (2 lb.), at 1s. 6d. ... ... | 0 | 4 | 6 |
| 4 | Glass stoppered bottles, 90 oz. (for lotions), at 7½d. ... ... ... ... ... ... | 0 | 2 | 6 |
| | | £5 | 16 | 1½ |

Surgical Dressings and Appliances.

| | | £ | s. | d. |
|---|---|---|---|---|
| 28 lb. | Boric lint, at 1s. 3d. ... ... ... ... | 1 | 15 | 0 |
| 56 ,, | Absorbent wool, at 9½d. ... ... ... | 2 | 4 | 4 |
| 28 ,, | Surgeons' lint, at 1s. 3d. ... ... ... | 1 | 15 | 0 |
| 1 | Store-box adhesive strapping on holland ... | 1 | 7 | 6 |
| 14 lb. | Gamgee tissue, at 1s. 7d. ... ... ... | 1 | 2 | 2 |
| 14 ,, | Cyanide wool, at 2s. ... ... ... ... | 1 | 8 | 0 |
| 144 yds. | Cyanide gauze, at 2d. ... ... ... | 1 | 4 | 0 |
| 14 lb. | Grey wool 2nd (for padding splints), at 6½d. | 0 | 7 | 7 |
| 12 yds | Oiled cotton, at 1s. 3d. ... ... ... | 0 | 15 | 0 |
| 6 ,, | English gutta percha (11 oz.), at 10s. ... | 0 | 7 | 0 |
| 12 ,, | Flannel domette (for bandages), at 7d. ... | 0 | 7 | 0 |
| 1 ,, | Spongio piline, at 13s. ... ... ... | 0 | 13 | 0 |
| 12 ,, | Opal cotton, at 1s. 9d. ... ... ... | 1 | 1 | 0 |
| 1 gr. | Grey calico bandages, 3 inch, at 18s. 6d. ... | 0 | 18 | 6 |
| ½ ,, | Grey open wove bandages, 3 inch, at 16s. 6d. | 0 | 8 | 3 |
| 1 cwt. | Tow, at 4d. ... ... ... ... ... | 1 | 17 | 4 |
| 2 doz. | Sponges (assorted), at 10s. ... ... ... | 1 | 0 | 0 |
| 2 | Ice bags... ... ... ... ... ... | 0 | 5 | 7 |
| 2 | Water pillows, 24 by 20 ... ... ... | 1 | 13 | 0 |
| 6 yds | Macintosh cloth, at 2s. 10d.... ... ... | 0 | 17 | 0 |
| 22 ,, | Macintosh cloth, $\frac{24}{24}$, at 4s. 6d. ... ... | 4 | 19 | 0 |
| 4 sets | Fracture boards ... ... ... ... | 3 | 12 | 0 |
| 2 | Bloxam cradles (with back and side splints) | 6 | 0 | 0 |
| 1 | Iron-framed canvas bed-rest... ... ... | 0 | 15 | 6 |
| 1 | Incline plane ... ... ... ... ... | 0 | 15 | 0 |
| 2 | Back leg splints ... ... ... ... | 0 | 14 | 0 |
| 1 | Back leg splint, long, for patellas ... ... | 0 | 8 | 0 |
| 1 | Bandaging machine ... ... ... ... | 0 | 18 | 0 |
| 2 | Round tin pans with handles (receivers for dressings) ... ... ... ... ... | 0 | 4 | 0 |
| 4 | Bright block tin boxes (for dressings) ... | 1 | 12 | 0 |
| 1 | Sawdust tin (for operating-room) ... ... | 0 | 2 | 6 |
| 1 | Brass lamp, with reflector and chimney ... | 0 | 3 | 6 |
| 1 | Tripod (for use in wards instead of crutches) | 0 | 11 | 0 |
| | | £42 | 0 | 9 |

Surgical Instruments.

| Quantity. | | £ | s. | d. |
|---|---|---|---|---|
| 1 | Scoop in metal handle ... ... ... | 0 | 6 | 6 |
| 6 | Scalpels in fluted metal handles at 2s. 6d... | 0 | 15 | 0 |
| 2 prs. | Dissecting forceps, 1 at 2s., 1 at 3s. ... | 0 | 5 | 0 |
| 20 ,, | Wells' forceps, N.P., at 5s. 6d. ... ... | 5 | 10 | 0 |
| 1 pr. | Vulsellum forceps, N.P. ... ... ... | 0 | 8 | 6 |
| 1 | Amputating knife, 9 in., in metal handles... | 0 | 12 | 6 |
| 1 | Amputating knife, 6in., in metal handles... | 0 | 9 | 6 |
| 4 | Bistouries, assorted, in fluted metal handles, at 4s. 6d. ... ... ... ... ... | 0 | 18 | 0 |
| 3 | Silver Probes, at 1s., 2s. 6d., 3s. 6d. ... | 0 | 7 | 0 |
| 1 | Steel director ... ... ... ... ... | 0 | 4 | 6 |
| 1 | Key's N.P. director ... ... ... ... | 0 | 4 | 6 |
| 2 | Blunt hooks in fluted metal handles, at 3s. 6d. ... ... ... ... ... | 0 | 7 | 0 |
| 1 pr. | Copper retractors ... ... ... ... | 0 | 4 | 0 |
| 1 | Esmarch's bandage and cord with chain, complete in tin case ... ... ... ... | 1 | 1 | 0 |
| 1 | Amputating saw in movable handle, N.P. | 1 | 1 | 0 |
| 1 pr. | Bone forceps, N.P. ... ... ... ... | 0 | 11 | 6 |
| 1 ,, | Lion forceps, N.P. ... ... ... ... | 0 | 10 | 6 |
| 6 | Sponge holders, at 4s. 6d. ... ... ... | 1 | 7 | 0 |
| 6 pkts | Hagedorn's assorted needles .. ... | 0 | 7 | 6 |
| 6 ,, | Straight triangular needles ... ... | 0 | 7 | 6 |
| 6 ,, | Intestine needles ... ... ... ... | 0 | 7 | 6 |
| 1 | Improved Hagedorn's needle holder ... | 0 | 15 | 0 |
| 1 doz. | Best black catheters with aseptic ends ... | 1 | 10 | 0 |

Surgical Instruments (*continued*).

| Quantity. | | £ | s. | d. |
|---|---|---|---|---|
| 1 doz. | I.R. catheters (best) ... ... ... ... | 0 | 18 | 0 |
| 1 | Potains aspirator, 2 trocars and stopcock in case ... ... ... ... ... ... | 3 | 3 | 0 |
| 1 | Thompson's bladder sound ... ... ... | 0 | 9 | 6 |
| 1 | Exploring trocar, in ivory case ... ... | 0 | 6 | 0 |
| 1 | Boyd's modified Volkman's scoop, N.P. ... | 0 | 7 | 6 |
| 3 | Parker's silver trachea tubes and two laryngotomy tubes ... ... ... ... | 3 | 12 | 6 |
| 3 | Trephines, fitting one handle, N.P. ... | 1 | 15 | 0 |
| 1 | Elevator, solid steel, N.P. ... ... ... | 0 | 4 | 6 |
| 1 pr. | Hoffmann's bone forceps, N.P. ... ... | 1 | 1 | 0 |
| 1 | Hey's saw ... ... ... ... ... | 0 | 4 | 6 |
| 1 | Clover's gas and ether apparatus, in case ... | 3 | 10 | 6 |
| 1 | Carter Braine's improved Junker's inhaler | 2 | 10 | 0 |
| 1 | Skinner's mask ... ... ... ... | 0 | 5 | 7 |
| 1 | Hewett's gag ... ... ... ... ... | 1 | 17 | 6 |
| 1 | Bloxam's chloroform drop bottle ... ... | 0 | 4 | 6 |
| 1 | Razor and strop ... ... ... ... | 0 | 5 | 6 |
| 1 pr. | Sinus forceps, N.P. ... ... ... ... | 0 | 5 | 6 |
| 1 | Silver and platinum caustic holder... ... | 0 | 13 | 6 |
| 3 prs. | Scissors ... ... ... ... ... ... | 0 | 11 | 6 |
| 1 pr. | Scissors, iridectomy ... ... ... ... | 0 | 6 | 0 |
| 1 ,, | Scissors, blunt-pointed curved ... ... | 0 | 7 | 6 |
| 1 | Higginson's syringe ... ... ... ... | 0 | 7 | 6 |
| 1 | 2 oz. brass syringe ... ... ... ... | 0 | 9 | 6 |
| 1 | Hypodermic syringe ... ... ... ... | 0 | 9 | 6 |
| 6 | Hanks horsehair ... ... ... ... | 0 | 6 | 0 |
| 6 | Hanks dry chromic catgut ... ... ... | 0 | 3 | 0 |
| 6 | Hanks plaited silk ... ... ... ... | 0 | 6 | 0 |
| 2 | Hanks Chinese twist ... ... ... ... | 0 | 2 | 0 |
| 6 | Hanks silkworm gut ... ... ... ... | 0 | 6 | 0 |
| 3 | Reels silver wire ... ... ... ... | 0 | 13 | 6 |
| 1 pr. | Dressing forceps ... ... ... ... | 0 | 5 | 6 |
| 1 | Finger saw ... ... ... ... ... | 0 | 3 | 0 |
| 1 | Solid steel gouge, N.P. ... ... ... | 0 | 5 | 0 |
| 1 | Scalpel case and brass racks for 12 ... | 0 | 3 | 6 |
| 1 | Urinary test stand and drawer, complete with test tubes, urinometer, spirit lamp, engraved glass bottles, pipette, &c ... | 1 | 15 | 6 |
| | | £47 | 5 | 1 |

Summary.

| | | £ | s. | d. |
|---|---|---|---|---|
| Furniture | Wards (eight beds) ... ... ... | 98 | 8 | 1 |
| | Operating-room and dispensary ... | 24 | 18 | 7 |
| | Matron's sitting-room ... ... | 51 | 6 | 2½ |
| | Nurses' sitting-room ... ... ... | 12 | 14 | 6 |
| | Matron's bed-room ... ... ... | 13 | 19 | 3 |
| | Three nurses' bed-rooms ... ... | 34 | 9 | 2 |
| | Servants' bed-room (for two) ... | 10 | 5 | 0 |
| | Kitchen and scullery... ... ... | 5 | 4 | 1 |
| Linen (wards and staff) £25 16s. 6d.—£12 17s. 6d. —£4 1s. 9½d. ... ... ... ... ... | | 42 | 15 | 9½ |
| China and glass ... ... ... ... ... | | 7 | 0 | 8½ |
| Ironmongery ... ... ... ... ... ... | | 25 | 18 | 6 |
| China and enamelled goods for wards ... | | 5 | 16 | 1½ |
| Surgical dressings and appliances ... ... | | 42 | 0 | 9 |
| Surgical instruments ... ... ... ... | | 47 | 5 | 1 |
| Sundries ... ... ... ... ... ... | | 39 | 11 | 1½ |
| | | £461 | 12 | 11½ |

